# lncRNA OR3A4 Promotes the Proliferation and Metastasis of Ovarian Cancer Through KLF6 Pathway

**DOI:** 10.3389/fphar.2021.727876

**Published:** 2021-10-27

**Authors:** Fangfang Guo, Jianan Du, Lingling Liu, Yawei Gou, Mingming Zhang, Wei Sun, Hongmei Yu, Xueqi Fu

**Affiliations:** ^1^ Edmond H. Fischer Signal Transduction Laboratory, College of Life Sciences, Jilin University, Changchun, China; ^2^ Department of Gynecology, Xinhua Hospital Affiliated to Dalian University, Dalian, China; ^3^ Department of Molecular Biology, College of Basic Medical Sciences Jilin University, Changchun, China; ^4^ Jilin Province Zebrafish Genetic Engineering Laboratory, Jilin Provincial Development and Reform Commission, Changchun, China; ^5^ Department of Blood Transfusion, China-Japan Union Hospital of Jilin University, Changchun, China

**Keywords:** ovarian cancer, lncRNA OR3A4, KLF6, inflammation, zebra fish

## Abstract

**Aim:** Ovarian cancer is a collaborative malignant tumor of the female reproductive system in clinical research. Some clinical studies have shown that OR3A4, which is a cancer-causing lncRNA, plays a major role in promoting the occurrence and development of a variety of tumors. And we also expressed the view that it expressed in ovarian tissue. However, the function of OR3A4 in ovarian cancer remains unclear.

**Methods and Results:** To further verify the function of lncRNA OR3A4 in ovarian cancer, we established the xenograft model in the zebra fish. In this study, cells transformed with OR3A4 shRNA plasmids were transplanted into the zebra fish, and the cell proliferation and migration ability were significantly reduced compared to the empty vector. While knocking out OR3A4, we further downregulated its expression by siRNA of KLF6. Our study found that the knocked out OR3A4 resulted in a decrease in cell proliferation and migration level, which can be found in the downregulated expression of KLF6. We also verify the relationship between OR3A4 and circulating tumor cells in the zebra fish xenograft model, the results indicate that lncRNA OR3A4 may be involved in the resistance of ovarian cancer to complain.

**Conclusion:** lncRNA OR3A4 promotes the proliferation and metastasis of ovarian cancer through the KLF6 pathway.

## Introduction

Ovarian cancer is a joint malignant tumor of the female reproductive system in clinical research (Jemal et al., 2007; Bowtell et al., 2015). In China, its incidence rate ranks the third after cervical cancer and endometrial cancer, which seriously threaten women’s health (Shi et al., 2017). At present, it has been observed that long non-coding RNA refers to RNA molecules with nucleotide length between 200 and 200,000 without translation function (Mercer et al., 2009; Zhang et al., 2013; Lorenzen and Thum, 2016). Its mechanism of action is mainly to interfere with the physiological activities of cells and play a role in the occurrence, progression, and metastasis of tumors by affecting the expression of downstream genes and interfering with the normal shearing of mRNA (Maass et al., 2014; Schmitt and Chang, 2016; Luo et al., 2018; Chen et al., 2019).

Long non-coding RNA OR3A4 is the member of the olfactory receptor family 3 and subfamily A (Shang et al., 2019). Studies have reported that the expression of OR3A4 can be detected in the peripheral blood of primary gastric cancer tissues and gastric cancer patients, and it has also been found that OR3A4 can promote the growth, invasion, metastasis, and tumorigenesis of gastric cancer cells *in vitro* and *in vivo* (Guo et al., 2016). This indicates that OR3A4 is a cancer-causing lncRNA that promotes tumor progression. Clinical studies have confirmed that it plays an important role in promoting the occurrence and development of a variety of tumors , such as gastric cancer, breast cancer, liver cancer, and lung cancer (Gutschner and Diederichs, 2012; Guo et al., 2016; Liu et al., 2017; Li et al., 2019a; Zhong et al., 2019). However, the expression and function of long non-coding RNA OR3A4 in ovarian cancer remains unclear.

Kruppel-like factor 6 (KLF6) is a member of the Kruppel-like factor family and is considered a tumor suppressor (Zhang et al., 2018). Research proves that KLF6 in human cancer mainly by p53 independence means an increased expression of p21 (Lang et al., 2013; D'Astolfo et al., 2008), makes the cell cycle of tumor cells stranded in the G_1_/S transition, and thus plays a role of inhibition of the proliferation of cancer cells, transfer, etc. In a variety of cancers, such as rectal cancer and liver cancer (Reeves et al., 2004; Watanabe et al., 2008), KLF6 can lead to a decreased protein expression level due to gene deletion or mutation, resulting in the occurrence and development of tumors. At present, studies have shown that KLF6 silencing in ovarian cancer can promote cell and tumor growth and blood vessel production, but whether it can promote apoptosis of ovarian cancer has yet to be further studied (DiFeo et al., 2006; Han and Gong, 2021; Zhang et al., 2021). Therefore, the role of long non-coding RNA OR3A4 and KLF6 in ovarian cancer, and whether they are related became the purpose of this study. By detecting the overexpression of long non-coding RNA OR3A4 in ovarian cancer tissues and ovarian cancer cells, this study found that the increased expression of OR3A4 could inhibit the expression of KLF6, and the knockdown of OR3A4 could inhibit the migration and invasion of ovarian cancer cells. In order to further prove the role of KLF6 in ovarian cancer, this study used siRNA technology to study the role of KLF6 in isolation-induced apoptosis of ovarian cancer cells, providing a theoretical basis for further revealing the role of KLF6 in the pathogenesis of ovarian cancer.

The results show that in ovarian cancer, lncRNA OR3A4 can inhibit the expression of KLF6 expression and, at the same time, promote the cell migration and invasion ability, and the declined KLF6 expression can also block the apoptosis induced by cisplatin. lncRNA OR3A4 promotes cell migration and invasion ability of important roles and molecular mechanism, provides a theoretical basis for the future of clinical trials and drug development, and provides a new potential therapeutic target and idea. In addition, studies have shown that inflammation and tumor progression are closely linked. It was reported that chronic inflammation was related to 15–20% of tumor occurrence, such as lung cancer, gastrointestinal tumors, and breast cancer are related to systemic inflammation (Tomita et al., 2011; Xu et al., 2017). Inflammation can upregulate tumor-causing cytokines and inflammatory mediators, inhibit tumor cell apoptosis, induce tumor angiogenesis, stimulate DNA damage, induce immunosuppressive microenvironment, and remodel the extracellular matrix (Coussens and Werb, 2002; Hanahan and Weinberg, 2011). Besides, studies have likewise reported the impact of antitumor drugs on the immune response, such as methotrexate (MTX) blocking the acute inflammatory immune response after wasp infection (Yadav et al., 2021). It suggests that the effects of inflammation and tumors are mutual. In this study, we have also observed that the administration of the antitumor drug cisplatin increases the migration and aggregation of neutrophils, which are among the most quickly responsive innate immune cells in inflammation. This model could be invoked as a basis for studying the correlation between immune cells and tumors in the further study.

## Materials and Methods

### Cells and Drugs

SKOV3 cell lines were obtained from the Boster Biological Technology Co., Ltd. (Wuhan, Hubei, China). SKOV3 cell lines were cultured in an RPMI-1640 medium supplemented with 10% fetal bovine serum (FBS) in a 37°C incubator containing 5% CO2. Cisplatin was purchased from Sigma (St. Louis, MO, United States).

### Cell Proliferation

For the CCK8 assay, a cell counting kit 8 (CCK8, Dojindo, Japan) was used according to the manufacturer’s instructions. SKOV3+EV, SKOV3+OR3A4 shRNA, and SKOV3+OR3A4 shRNA + KLF6siRNA cells were respectively placed in a 1640 medium for 24 h, followed by incubation in 96-well plates at a density of 5 × 103 cells/well, and cultured for 24, 48, and 72 h, respectively. At the appropriate time points, 10 μL of CCK8 reagent was added to each well and mixed uniformly. Then all cells were incubated for another 3 h, and the optical absorbance at 450 nm was measured with an ELISA plate reader (Thermo Fisher, United States).

### Cell Migration

SKOV3 cell migration was analyzed using a wound healing assay. A culture insert was set into a 12-well plate, following which SKOV3+EV, SKOV3+OR3A4 shRNA, and SKOV3+OR3A4 shRNA + KLF6siRNA cells were counted and seeded (8 × 105) into each well containing the culture insert with 70 μL of medium and incubated for 24 h. Suction was utilized to draw out the medium from the insert, and the insert was gently removed using sterile tweezers, followed by the addition of 2 ml of medium to each well of the plate. Finally, cell migration was observed for 0–8 h.

### Western Blotting

Western blotting was done using the standard protocol. In brief, the samples were lysed and denatured in 5× sample buffer. The same amount of proteins was separated on a 10% SDS–polyacrylamide gel and transferred onto a nitrocellulose membrane. The nitrocellulose membrane was incubated with 5% non-fat milk in Tris-buffered saline (150 mM NaCl, 20 mM Tris–HCl, pH7.4) with a primary antibody overnight at 4°C. After washing, the membrane was further incubated with a secondary antibody for 45 min, and proteins were detected with an ECL detection system. ImageJ software was used to measure the band intensity.

### Zebra Fish Xenograft Model Experiment

Cells of shRNA were transfected for 24 h, and empty vectors were collected and washed three times with HBSS. The labeled cells were stained with the lipophilic fluorescent tracking dye CM-DiI. The cells were seen at 37°C for 5 min and then at 4°C for 15 min. Finally, the cells were washed three times with HBSS. The labeled cells were used only for injection. The zebra fish embryos at 48 hpf (hours post fertilization) were fixed using a low–melting point agarose gel; the tumor cell suspension is loaded into a capillary needle and injected into the protriptyline space with the amount of 300–400 cells in one strip. The zebra fish injected with the tumor cells is cultured in a 34°C incubator. The cells were transplanted for observation under a microscope on the second day (1 day post-injection, dpi). Zebra fish larvae with similar tumor cell sizes were selected and cultured in an incubator at 34°C for 96 h (4 dpi). Then they were fixed with the low–melting point gel and imaged using a fluorescence stereomicroscope (Olympus MVX10, Japan), with the magnifying power of ×3.2. The images were created with ImageJ software, and the statistical analysis was performed by GraphPad Prism 8 software.

### Drug Screening in Zebra Fish

After the zebra fish xenograft model was established, the zebra fish larvae with similar tumor cell mass sizes were selected for drug screening for 1 day. The drug concentration of 0.6 mg/ml was added to the zebra fish culture solution and administered by immersion. Fresh culture solution containing the drug was replaced every 24 h and cultured in an incubator at 34°C. The drug was administered continuously for 3 days (4 dpi), respectively. The methods of microimaging, picture processing, and statistical analysis were the same as before.

### Data Analysis

Group data were presented as mean ± SE. The unpaired *t*-test was used to compare between groups. Multiple group means were compared by ANOVA, followed by the least significant difference (LSD) post hoc *t-*test.

## Results

### Correlation Between KLF6 and Ovarian Cancer and Pathway Enrichment Analysis

Based on the TCGA PanCancer Atlas database of the cBioPortal website, we obtained the relationship between KLF6 and the incidence of various cancers. The alteration frequency of KLF6 in bladder cancer was the highest, which had a total of 8.76% alteration in 411 cases. Followed by ovarian cancer, there was 5.14% alteration of KLF6 in 584 cases. Among them, 0.17% were mutations, 0.17% were fusions, and 4.29% were amplification. Then we evaluated the effect of the KLF6 expression level on the survival of ovarian cancer patients through the TCGA OV database of the UALCAN Website. We found that when the KLF6 gene is highly expressed, the probability of patients surviving more than 2,000 days is significantly lower than that of the KLF6 medium- to low-expression group, which indicates that the expression level of KLF6 may be negatively correlated with the survival rate of ovarian cancer (*p* < 0.05).

**FIGURE 1:**
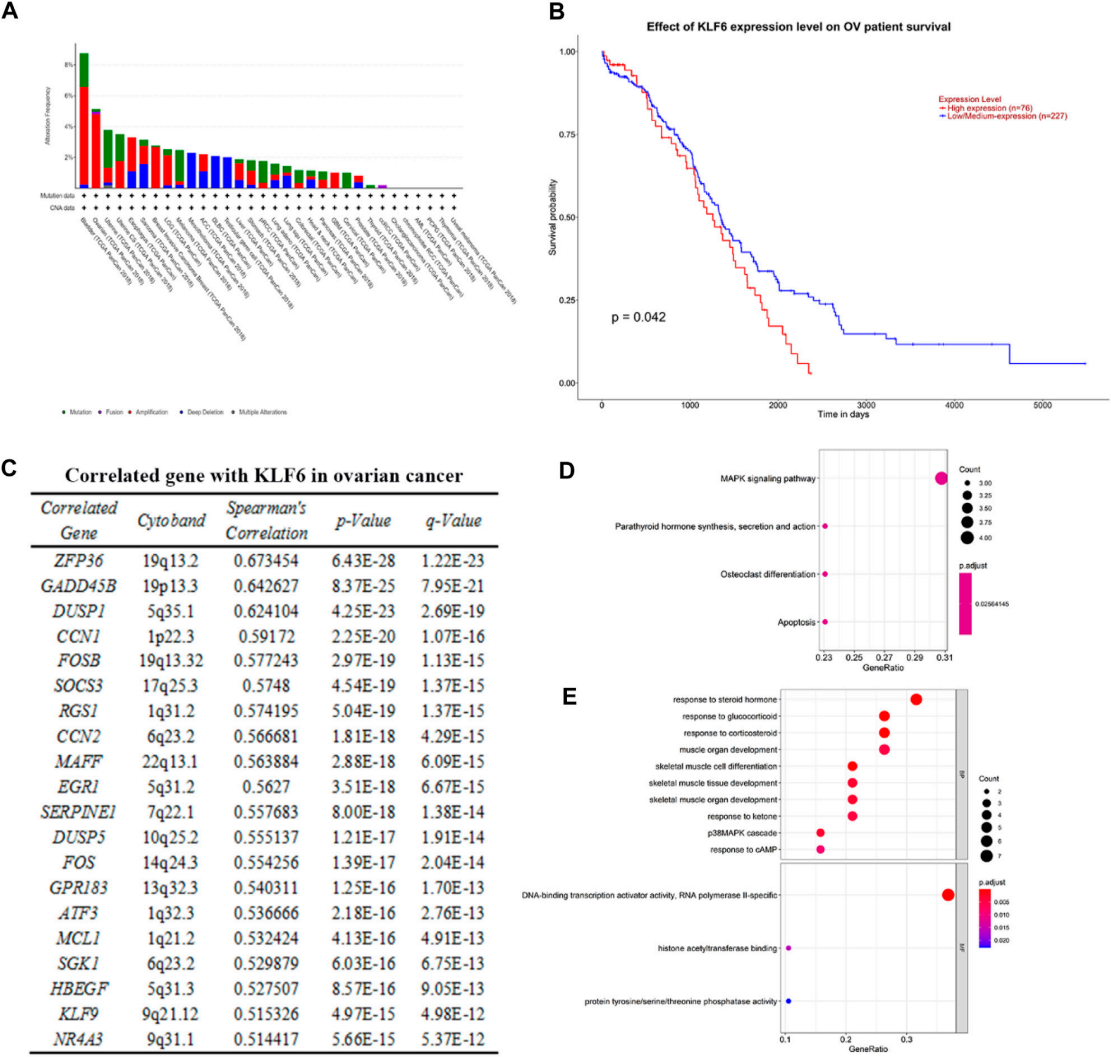
Correlation between KLF6 and ovarian cancer and pathway enrichment analysis. **(A)** Using the TCGA PanCancer Atlas database of cBioPortal, the alteration frequency of KFL6 in various cancer was obtained. **(B)** Effect of the KLF6 expression level of ovarian cancer patient survival based on the TCGA OV database of UALCAN. **(C)** Co-expressed genes with KLF6 in ovarian cancer samples according to the mRNA expression profiles of the TCGA database. **(D, E)** KEGG and GO analysis of modular genes by the R package of clusterProfiler. The enrichment file is in KEEG. and GO. txt.

After evaluating the correlation between KLF6 and ovarian cancer, we obtained 20 genes co-expressed with KLF6 in ovarian cancer samples based on the TCGA mRNA expression profiles on the cBioPortal Website. Subsequently, enrichment of co-related genes was analyzed by R language. The GO analysis results showed that these genes were predominantly enriched in response to hormones such as steroid hormones, glucocorticoid, and corticosteroid; muscular organ development; skeletal muscle cell differentiation; p38MAPK signal cascade; and other biological process (BP). Molecular function (MF) is mainly enriched in the activity of RNA polymerase II–specific DNA-binding transcription activator, histone acetyltransferase binding, and protein tyrosine/serine/threonine phosphatase activity. The KEGG pathway of these 20 genes is significantly enriched in the MAPK signaling pathway, parathyroid hormone synthesis, secretion and action, osteoclast differentiation, and apoptosis (Figure 1).

### Knocked Out of lncRNA OR3A4 Resulted in Decreased Proliferation and Migration Levels of SKOV3 in Zebra Fish Xenograft Model

**FIGURE 2:**
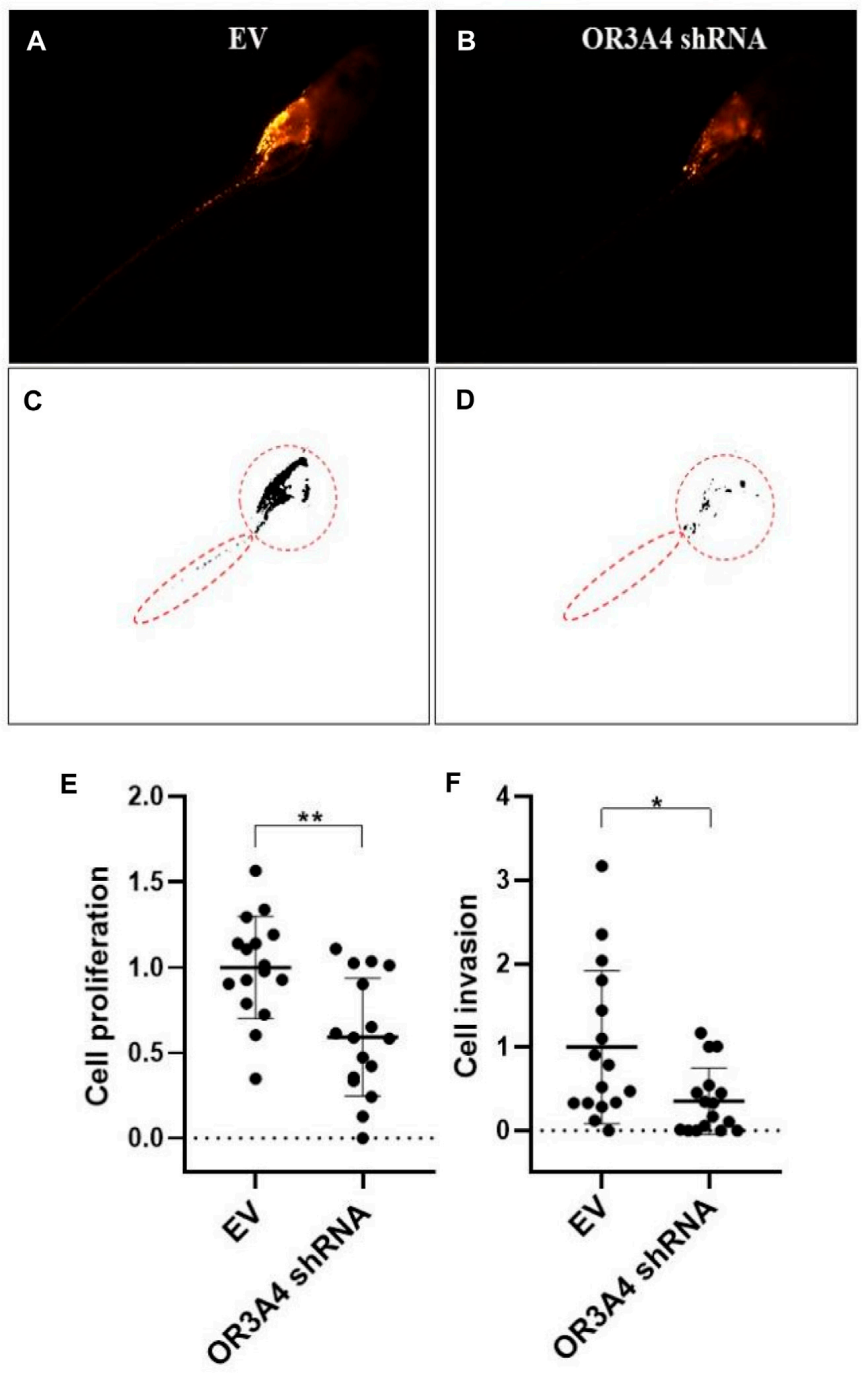
Knocked out of lncRNA OR3A4 in the zebra fish xenograft model resulted in decreased proliferation and migration of SKOV3 **(A, B)** Representative fluoroscopy image at 4 days after transplantation of the empty vector (EV) transfected with shRNA and the SKOV3 cells transfected with the lncRNA OR3A4 shRNA plasmid into the protriptyline space of zebra fish larvae at 3d after fertilization**(C, D)** Fluorescent segment images corresponding to those pictures. The signals in the circles (transplant sites) are employed to the statistical analysis of cell proliferation, and the signals in the oval circles (migrated trunk sites) are used for the statistical analysis of cell migration **(E, F)** Statistical analysis of the effects of transfection of OR3A4 shRNA plasmid on the proliferation and migration of SKOV3 in the zebra fish xenograft model. *p < 0.05, **p < 0.01, n = 16.

To further verify the function of lncRNA OR3A4 on SKOV3 *in vivo*, we established the xenograft model in the zebra fish. The zebra fish xenograft model has been used in tumor-related research in recent years mainly because of its high efficient transplantation and intuitive and rapid observation. In this study, the cells transformed with OR3A4 shRNA plasmids were transplanted into the protriptyline space around the yolk sac of zebra fish embryos at 3 days post-fertilization (3 dpf). Then we imaged and analyzed the SKOV3 proliferation and migration at 4 days after transplantation. It was noted that after the knockdown of OR3A4 in SKOV3 cells, the cell proliferation and migration ability were significantly reduced compared with the empty vector (Figure 2).

### KLF6 Mediated the Proliferation and Migration of lncRNA OR3A4 in SKOV3

**FIGURE 3:**
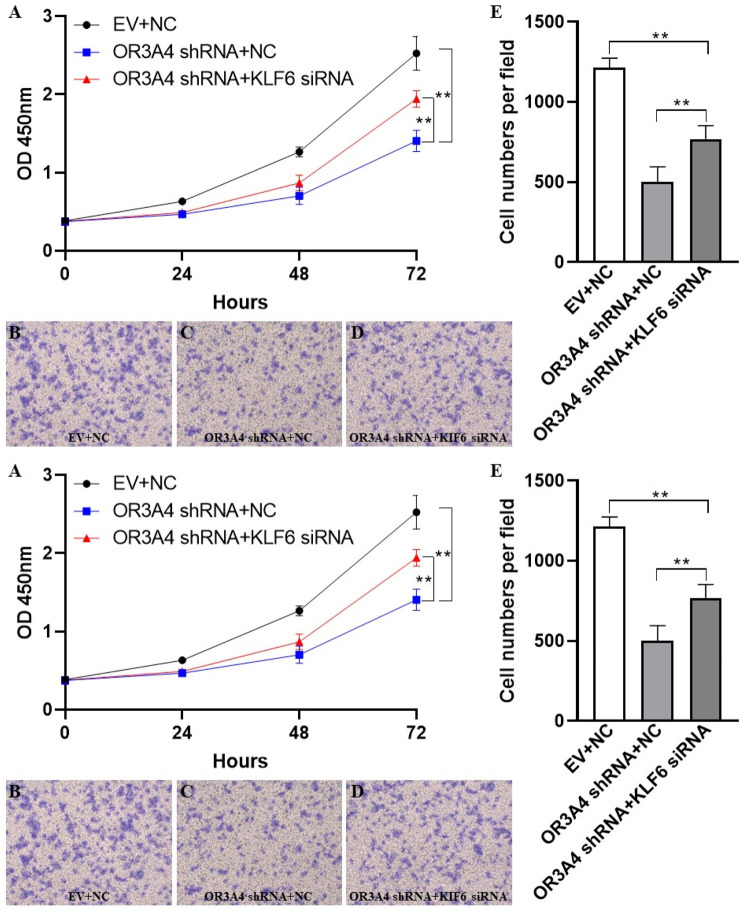
KLF6 mediated the proliferation and migration of lncRNA OR3A4 in SKOV3 **(A)** Knocking out of OR3A4 and the downregulation of KLF6 expression can rescue the proliferation of SKOV3 cells **(B, E)** Knocked out OR3A4 while downregulated KLF6 saved the migration level of SKOV3 cells **(B–D)** Typical pictures of transwell experiments.

To study the regulation of proliferation and migration of SKOV3 by lncRNA OR3A4 through the KLF6 pathway, we verified by cell rescue experiments. While knocking out OR3A4, we further downregulated its expression by siRNA of KLF6. Our study found that knocked out OR3A4 resulted in a decrease in cell proliferation and migration levels, which can be found by downregulating the expression of KLF6 (Figure 3). The result stated that OR3A4 could regulate the proliferation and migration of SKOV3 cells by downregulating the expression of KLF6.

### Knocked Out OR3A4 in SKOV3 Cells Enhanced the Antitumor Effect of Cisplatin

To study the effect of lncRNA OR3A4 on cisplatin resistance of SKOV3 cells, we treated with cisplatin, and on this basis, downregulated the expression of OR3A4, and then detected the cell proliferation and migration level. Studies have shown the same.

**FIGURE 4:**
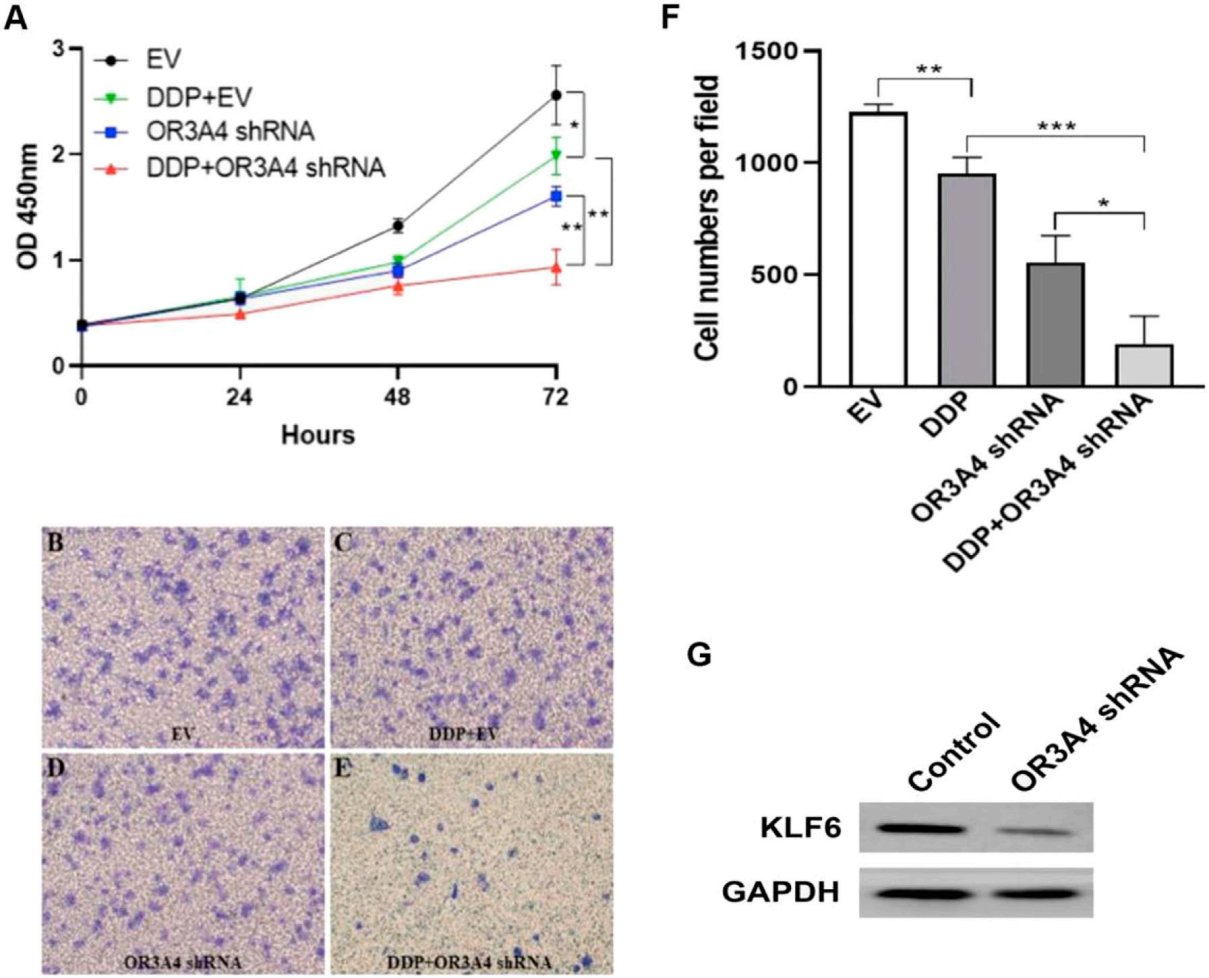
Knockdown of OR3A4 in SKOV3 cells enhanced the antitumor effect of cisplatin **(A)** The proliferation level of cells processed by DDP and simultaneously knocked down OR3A4 was significantly lower than those treated individually **(B–F)** The migration level of cells treated with DDP while knocking down OR3A4 was considerably lower than those treated individually **(B–E)** The representative images of transwell experiments **(F)** The statistical analysis of cell migration **(G)** KLF6 expression in SKOV3 cells while knocking down OR3A4. *p < 0.05, **p < 0.01, ***p < 0.001.

Proliferation and migration were significantly reduced after the concurrent use of circulating tumor cells, and the expression of OR3A4 was downregulated compared with that after a single treatment (Figure 4). The results indicate that lncRNA OR3A4 may be involved in the resistance of SKOV3 to cisplatin.

### The Zebra Fish Xenograft Model Verified that the Knockdown of lncRNA OR3A4 Enhanced the Tumor Inhibition Effect of Cisplatin

To further verify the relationship between lncRNA OR3A4 and cisplatin in the zebra fish xenograft model, we first tested the treated concentration of cisplatin in zebra fish. Because there may be differences between the soaking concentration and the concentration in zebra fish larvae, we started the experiment according to the general concentration of the cells of the experiment. We determined the appropriate concentration of cisplatin treatment according to the development of zebra fish larvae. We found that the concentration of cisplatin equal to or higher than 0.8 mg/ml had a significant effect on the development and survival of zebra fish, whereas when the concentration of cisplatin was equal to or lower than 0.6 mg/ml, the development of zebra fish larvae was unaffected.

**FIGURE 5:**
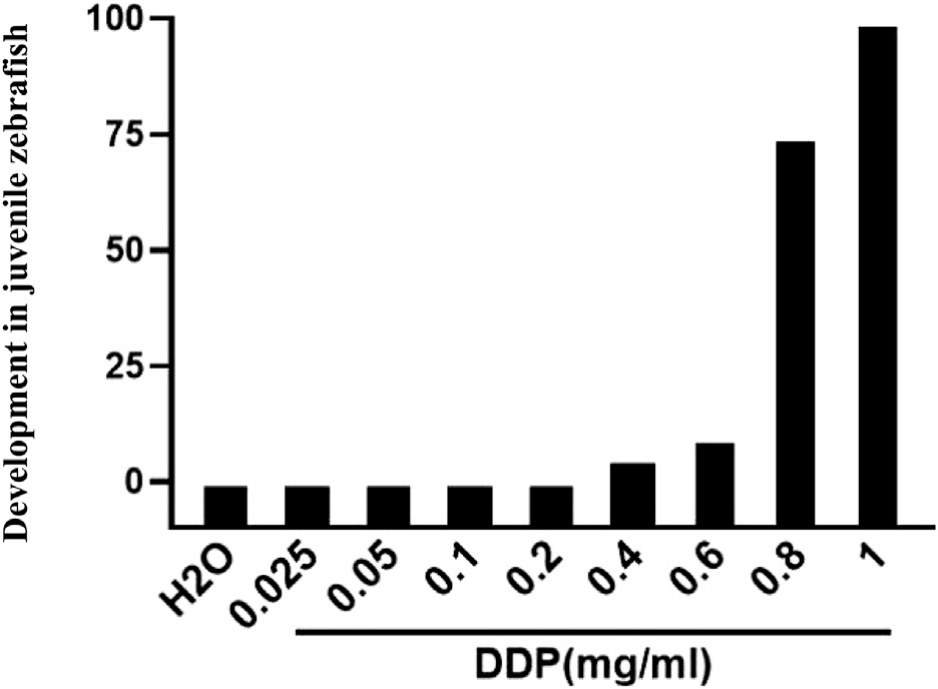
Impacts of different cisplatin concentrations on the overall development in juvenile zebra fish (3–6 dpf).

After determining the cisplatin treatment concentration in zebra fish larvae, we further performed the study based on the zebra fish xenograft model. Similar to the results from the cell experiment, we found that the proliferation and migration of tumor cells after simultaneous treatment with cisplatin and OR3A4 knockdown were significantly lower than those of a single treatment (Figure 5).

**FIGURE 6:**
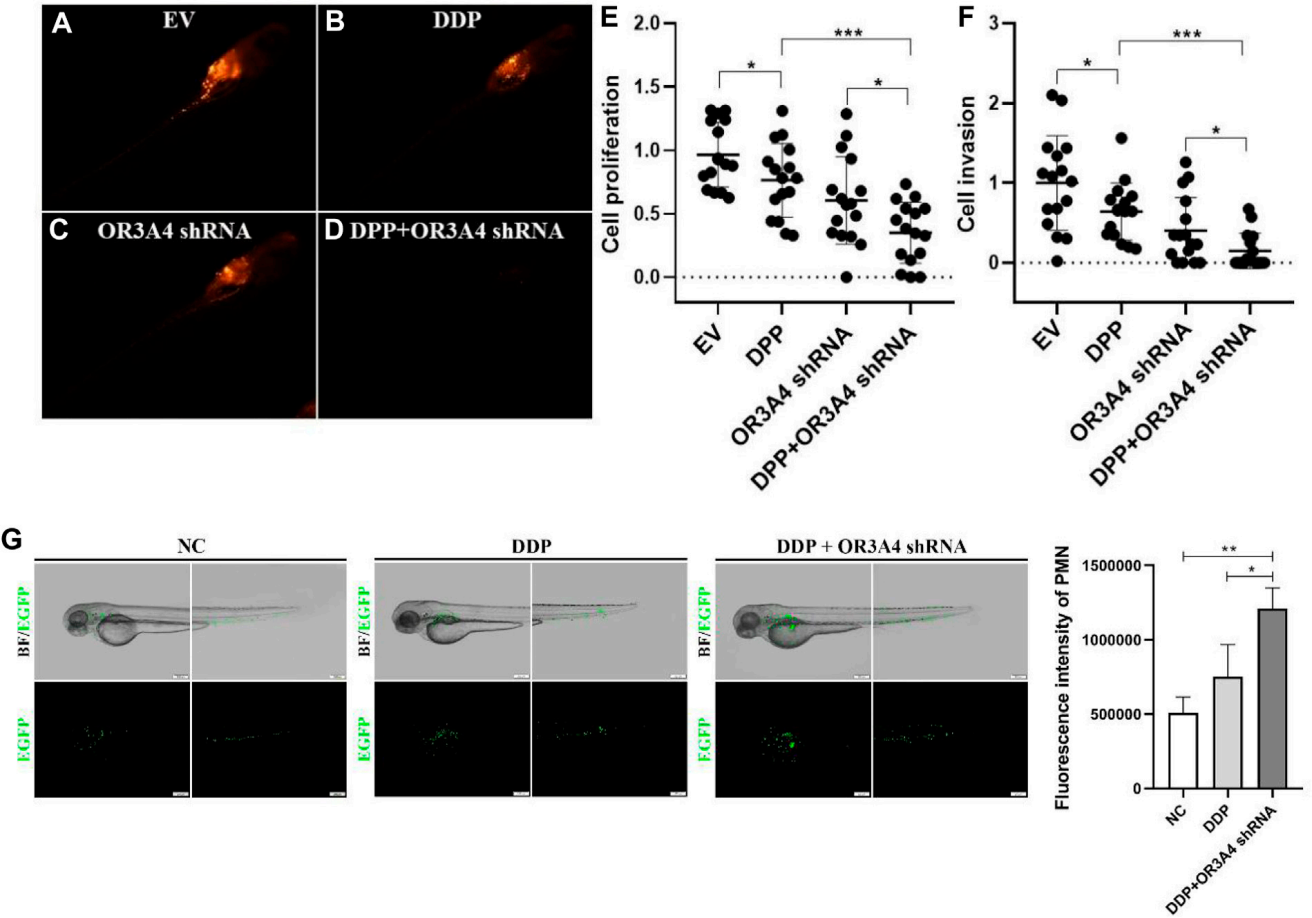
Concurrent treatment with DDP and knockdown of OR3A4 enhanced the tumor inhibition of cisplatin in the zebra fish xenografts model **(A–D)** The representative imaging of zebra fish larvae treated with DDP, knockout OR3A4, and concurrent treatment **(E–F)** Statistical effects of DPP, knockdown of OR3A4, and concurrent treatment on the proliferation and migration of SKOV3 cells in a zebra fish xenograft model **(G)** The representative images of the effects on the migration and aggregation of neutrophils in zebra fish larvae treated with DDP and knocked out OR3A4. *p < 0.05, ***p < 0.001, *n = 16.*

In addition, we used neutrophil EGFP-labeled juvenile zebra fish at 3 dpf to detect the effect of cisplatin treatment and OR3A4 knockdown on the immune cells. The results showed that compared with the DDP treatment alone, the migration and aggregation of neutrophils in zebra fish larvae after cisplatin combined with OR3A4 knockdown were significantly enhanced (Figure 6).

## Discussion

lncRNA is an important regulatory factor in the occurrence and development of ovarian cancer (Gutschner and Diederichs, 2012; Zhan et al., 2018; Long et al., 2019), which can be invoked as a potential biomarker and therapeutic target for ovarian cancer. For instance, the silencing of lncRNA MNX1-AS1 can inhibit the proliferation and migration of ovarian cancer cells (Gao et al., 2019), and lncRNA MNX1-AS1 may become a potential target of ovarian cancer. lncRNA BACE1-AS can inhibit the proliferation and invasion of ovarian cancer stem cells and can be utilized as a new target for ovarian cancer treatment (Chen et al., 2016). lncRNA HOXA1 can promote the proliferation and migration of ovarian cancer cells, which is closely linked to the prognosis of the ovary (Li et al., 2019b). Downregulation of lncRNA SPRY4-IT1 can promote the metastasis of ovarian cancer cells by regulating epithelial–mesenchymal transition (Li et al., 2017; Yu et al., 2017). lncRNA ElncRNA1, an oncogene in the proliferation of epithelial ovarian cancer cells, was substantially upregulated by estrogen (Qiu et al., 2017).

Krüppel-like factor 6(KLF6) is a member of the Krüppel-like factor family. It has been proved that KLF6 plays the role of tumor suppressor in various tumors by regulating diverse biological processes (Narla et al., 2001; Reeves et al., 2004). Studies have shown that the mechanism of KLF6 inhibiting tumor is to increase the expression of p21 through the p53-independent pathway, thus inhibiting the occurrence and proliferation of cancer cells. At present, lncRNA OR3A4 in the occurrence and development of ovarian cancer is difficult to identify and the role of KLF6 as a tumor suppressor in ovarian cancer is also unknown. Therefore, this study aims to take lncRNA OR3A4 and KLF6 as research objects and try to explore their roles in ovarian cancer cells.

To study that lncRNA OR3A4 regulates the proliferation and migration of SKOV3 through the KLF6 pathway, we verified it by saving experiments in cells. The results showed that the knockdown of OR3A4 resulted in a decrease in cell proliferation and migration levels, which could be found by downregulating KLF6 expression. To further verify the relationship between lncRNA OR3A4 and cisplatin in the zebra fish xenograft model, we first tested the treated concentration of cisplatin in zebra fish. Because there may be differences between the soaking concentration and the concentration in zebra fish larvae, we started the experiment according to the general concentration of cells in experiment. We determined the appropriate concentration of cisplatin treatment according to the development of zebra fish larvae.

We found that the concentration of cisplatin equal to or higher than 0.8 mg/ml had a significant effect on the development and survival of zebra fish, whereas when the concentration of cisplatin was equal to or lower than 0.6 mg/ml, the development of zebra fish larvae was unaffected. Therefore, we used 0.6 mg/ml *in vivo* cisplatin treatment in zebra fish. Further studies have the responsibility for determining whether resistance to lncRNA OR3A4 is mediated by KLF6.

Moreover, cisplatin treatment combined with the knockdown of lncRNA OR3A4 enhanced tumor suppression, which may be related to the activation of innate immune cells, especially neutrophils. In this study, we first detected the role of neutrophils after tumor drug administration, which is a rapidly responsive cell in immunity. In further research, we will also study the role of macrophages, NK cells, T cells, B cells, and other immune cells in tumor killing.

## Data Availability

The original contributions presented in the study are included in the article/supplementary material; further inquiries can be directed to the corresponding authors.
